# Editorial: Ubiquitin and Ubiquitin-Relative SUMO in DNA Damage Response

**DOI:** 10.3389/fgene.2017.00188

**Published:** 2017-11-27

**Authors:** Kristijan Ramadan, Ivan Dikic

**Affiliations:** ^1^Department of Oncology, CRUK/MRC Oxford Institute for Radiation Oncology (MRC), Oxford University, Oxford, United Kingdom; ^2^Institute of Biochemistry II, School of Medicine, Goethe University, Frankfurt, Germany; ^3^Molecular Signaling Unit, Buchmann Institute for Molecular Life Sciences, Goethe University, Frankfurt, Germany

**Keywords:** DNA damage response, ubiquitin, ubiquitination, SUMO, sumoylation, cancer, genome stability

Ubiquitin (UB) is a small inactive peptide which dramatically changes the fate of ubiquitinated proteins when enzymatically activated and covalently attached to proteins in the process known as ubiquitination (Ciechanover et al., 1984). UB was initially discovered as a signal for UB-dependent protein degradation by the proteasome system in the late 1970s and early 1980s (Ciechanover et al., 1980, 1984; Hershko et al., 1980). However, it is now clear that the cellular role of ubiqutination is much more complex than initially thought (Grabbe et al., 2011). Ubiquitination is the most complex posttranslational modification (PTM) that regulates virtually all cellular processes (Komander, 2009; Heride et al., 2014; Swatek and Komander, 2016). Avram Hershko, Aaron Ciechanover and Irwin A. Rose were awarded with the Nobel Prize in Chemistry for 2004 for the discovery of the UB-mediated protein degradation (proteolysis) (Kresge et al., 2006). This award tremendously boosted scientific curiosity towards UB and ubiqutination as can be demonstrated by there currently being more than 70,000 Pubmed research articles on ubiqutination, compared to less than 12,000 before 2004. There are several ubiquitin like modifiers (Welchman et al., 2005), but SUMO is the best investigated one in DNA damage response (Schwertman et al., 2016). Therefore, this issue is focusing on UB and its main relative SUMO.

The DNA damage response (DDR) has been defined as a multifaceted network of cellular pathways that are activated after DNA damage (Jeggo et al., 2016). Various DNA lesions activate the DDR, which first senses DNA damage and then transduce this signal to downstream effectors that consequently govern a robust cellular response visualized as cell cycle arrest, DNA repair and/or apoptosis (Jackson and Bartek, 2009). The discovery of the cellular toolbox for repairing damaged DNA was commemorated in 2015 when the Nobel Prize in Chemistry was awarded to Tomas Lindahl, Paul Modrich and Aziz Sancar (Lindahl et al., 2016). The DDR is composed of hundreds of different proteins, the function of which needs to be spatiotemporally orchestrated, and this occurs via various PTMs. In the last decade the PTMs, ubiquitination and SUMOylation, have emerged as the essential and most critical PTMs in the regulation of the DDR (Jackson and Durocher, 2013; Schwertman et al., 2016). Defects in the components of UB system in DDR are associated with many human diseases, including cancer and accelerated ageing. Thus, we decided to systematically review advances in this relatively young field, and to cover its role in DNA replication, DNA repair and mitosis. Our intention is to invite the most prominent scientists in the field, together with a selection of young and promising scientists, and give them the opportunity to summarize our current knowledge of UB and SUMO in the regulation of the DDR. The main goal is to share current visions and directions that will shape the priorities in this field for the next 10 years. The majority of invited scientists gladly contributed to this special issue, either by writing review articles or reviewing submitted manuscripts. Thus, we would like to thank them all for their enormous and professional contribution to the issue.

Helle Ulrich (Institute of Molecular Biology - Mainz) and her group highlighted that ubiquitination and SUMOylation control all aspects of DNA replication, from its initiation, elongation and termination, and not only translesion DNA synthesis as was initially proposed (Garcia-Rodriguez et al.). The group of Simone Sabbioneda (National Research Centre - Pavia) discussed how UB and SUMO control DNA damage tolerance, the last line of defense that allows completion of DNA replication in the presence of an unrepaired template. They focused on post-replication repair, the mechanism cells use to bypass highly distorted templates caused by damaged bases (Cipolla et al.). Jacqueline Jacobs (Netherlands Cancer Institute) and her group demonstrated that UB and SUMO play an essential role in both telomere maintenance and protection, but are also key contributors for the cellular response to dysfunctional telomeres (Yalçin et al.). Besides the physiological role of the UB and SUMO pathways in DNA replication and telomere function, this issue also covers the majority of DNA repair pathways. Thus, Coleman and Huang (New York University School of Medicine) nicely summarized how SUMOylation plays a major role in fine-tuning of the Fanconi-Anemia Pathway, the main pathway for repairing DNA interstrand crosslinks. The group of Hanspeter Naegeli (University of Zurich) highlighted the essential importance of ubiquitination, SUMOylation but also Neddylation in the regulation of nucleotide excision repair, the main mechanism that protects us from UV-light (Rüthemann et al.). Smeenk and Mailand (University of Copenhagen) gave us comprehensive overview of UB and SUMO in the repair of DNA double strand break (DSB) repair, the most cytotoxic DNA lesion. Their work clearly demonstrates how ubiquitination and SUMOylation are highly sophisticated and complex PTMs in the DDR. Harding and Greenberg (University of Pennsylvania) presented an additional perspective on DSB repair, with a special focus on nuclear architecture, chromatin dynamics and chromatin organization in DSB repair and how UB and SUMO control and connect these processes. Himmels and Sartori (University of Zurich) went even deeper in the understanding of DSB repair and described how UB and SUMO regulate DNA-end resection, the initial step in DSB repair. Interestingly, they concluded that the UB pathway in DNA-end resection is mostly linked to protein degradation processes, where SUMO acts as an intermolecular “glue” in modulating protein-protein or protein-DNA interactions required for homologous recombination rather than specifically affecting the activity of individual proteins. Dantuma and Pfeiffer (Karolinska Institute, Stockholm) discussed how the E3-UB and E3-SUMO ligases are recruited to sites of DNA damage and the importance of the spatiotemporal relationship among different DNA repair proteins and PTMs. Pellegrino and Altmeyer (University of Zurich) nicely explained how the crosstalk between ubiqutination, SUMOylation and PARylation, another PTM that also forms a chain signal (PAR), regulate genome stability. Pinto-Fernandez and Kessler (University of Oxford) demonstrated the importance of inactivation of the ubiquitin signal in the DDR in their summary of how deubiquitinating enzymes counteract DDR-related ubiquitination. Beside the essential role of UB, SUMO and PAR in the spatiotemporal recruitment of different DNA replication and repair proteins at sites of DNA damage, the group of Thorsten Hoppe (University of Cologne) discussed how protein disassembly is equally as important as protein recruitment for genome stability (Franz et al.). The disassembly of proteins from chromatin is mostly orchestrated by the ubiquitin-dependent AAA+ATPase p97/Cdc48, also known as VCP in humans, that serves as the unfoldase and segregase to remove ubiquitinated proteins (Vaz et al., 2013; Bodnar and Rapoport, 2017). Ferrari and Gentili (University of Zurich) described the involvement of the DDR in the G2/M-checkpoint and mitosis and how these two processes are regulated by PTMs. In addition to molecular mechanisms of UB and SUMO in DDR and genome stability, this issue also contains one technical article, which helps us to better understand how to quantitatively investigate UB and SUMO pathways in DDR. Heidelberger et al. (Institute of Molecular Biology - Mainz and Goethe University, Frankfurt) described mass spectrometry-based approaches for quantitative analyses of site-specific protein ubiqutination in the context of the DDR.

## Concluding remarks

By reading these outstanding articles one can easily conclude that all authors strongly emphasize the promising therapeutic potential that targeting two PTMs- ubiquitination and SUMOylation- as well as other components of the DDR, has for cancer therapy (Hoeller and Dikic, 2009; Shen et al., 2013; Bassermann et al., 2014). As editors, we share the opinion of the authors. The best examples are the recently approved PARP inhibitor Olaparib for the treatment of BRCA-deficient cancers and the proteasome inhibitor Bortezomib for treating B-cell lymphomas. Indeed, many pharmaceutical companies have been intensively working on the inhibitors that target the components of the DDR and UB system. Many of these inhibitors are currently in pre-clinical or clinical trials (Deshaies, 2014). We would be extremely happy if this special issue helps researchers to better understand the involvement of the UB and SUMO systems in the DDR. We also believe that the knowledge gathered here will help scientists and pharmaceutical companies to better understand how to utilize the enormous potential of the UB and SUMO system in DDR for cancer therapy. Last but not least, we would like to dedicate this special issue on UB and SUMO in the DDR to Prof Stefan Jentsch, who passed away recently. As a postdoc in Alexander Varshavsky laboratory, Stefan was the first to discover the link between the UB-system and DDR (Jentsch et al., 1987). During his independent scientific career Stefan's discoveries have shaped the field of UB, SUMO, and DDR (Hoppe and Branzei, 2017).

## Author contributions

All authors listed have made a substantial, direct and intellectual contribution to the work, and approved it for publication.

## Conflict of interest statement

The authors declare that the research was conducted in the absence of any commercial or financial relationships that could be construed as a potential conflict of interest. The reviewer PB declared a past co-authorship with one of the authors ID to the handling Editor.
